# Crystal structure of an unknown solvate of dodecakis­(μ_2_-alaninato-1:2κ^2^ *O*:*N*,*O*)cerium(III)hexa­nickel(II) aqua­tris­(hydroxido-κ*O*)tris­(nitrato-κ^2^ *O*,*O*′)cerate(III)

**DOI:** 10.1107/S2056989015017132

**Published:** 2015-09-17

**Authors:** Stanislav I. Bezzubov, Vladimir D. Doljenko, Andrei V. Churakov, Irina S. Zharinova, Yuri M. Kiselev

**Affiliations:** a Institute of General and Inorganic Chemistry, Russian Academy of Sciences, Leninskii prosp. 31, Moscow 119991, Russian Federation; b Department of Chemistry, M.V. Lomonosov Moscow State University, Leninskie Gory 1/3, Moscow 119991, Russian Federation

**Keywords:** crystal structure, cerium complex, l-alaninate ligand, SQUEEZE procedure

## Abstract

The chiral title compound, [CeNi_6_(C_3_H_6_NO_2_)_12_][Ce(NO_3_)_3_(OH)_3_(H_2_O)], comprises a complex heterometallic Ni/Ce cation and a homonuclear Ce anion. Both the cation and anion exhibit point group symmetry 3. with the Ce^III^ atom situated on the threefold rotation axis. The cation metal core consists of six Ni^II^ atoms coordinated in a slightly distorted octa­hedral N_2_O_4_ configuration by N and O atoms of 12 deprotonated l-alaninate ligands exhibiting both bridging and chelating modes. This metal–organic coordination motif encapsulates one Ce^III^ atom that shows an icosa­hedral coordination by the O-donor atoms of the l-alaninate ligands, with Ce—O distances varying in the range 2.455 (5)–2.675 (3) Å. In the anion, the central Ce^III^ ion is bound to three bidentate nitrate ligands, to three hydroxide ligands and to one water mol­ecule, with Ce—O distances in the range 2.6808 (19)–2.741 (2) Å. The H atoms of the coordinating water mol­ecule are disordered over three positions due to its location on a threefold rotation axis. Disorder is also observed in fragments of two l-alaninate ligands, with occupancy ratios of 0.608 (14):0.392 (14) and 0.669 (8):0.331 (8), respectively, for the two sets of sites. In the crystal, the complex cations and anions assemble through O—H⋯O and N—H⋯O hydrogen bonds into a three-dimensional network with large voids of approximately 1020 Å^3^. The contributions of highly disordered ethanol and water solvent mol­ecules to the diffraction data were removed with the SQUEEZE procedure [Spek (2015 ▸). *Acta Cryst.* C**71**, 9–18]. The given chemical formula and other crystal data do not take into account the unknown amount of these solvent mol­ecules.

## Related literature   

Mol­ecular magnets based on 3*d*–4*f* heterometallic constituents can be prepared easily by self-assembling of simple building blocks such as *d*-metal amino acid salts and lanthanide nitrates (Peristeraki *et al.*, 2011 ▸; Yukawa *et al.*, 2005 ▸; Igarashi *et al.*, 2000 ▸). For an icosa­hedral coordination environment observed in similar compounds, see: Peristeraki *et al.* (2011 ▸); Zhang *et al.* (2004 ▸). For background to and application of the SQUEEZE procedure, see: Spek (2015 ▸).
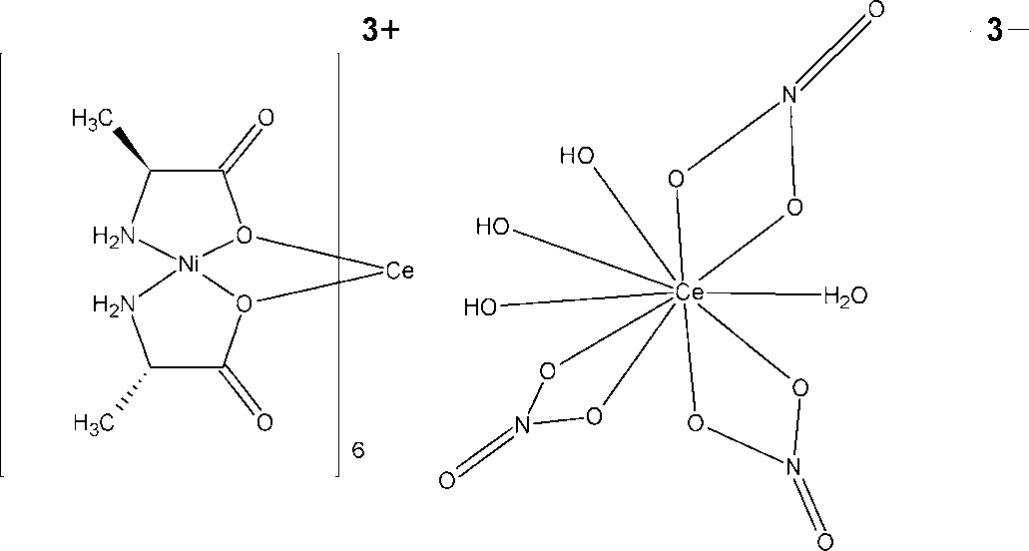


## Experimental   

### Crystal data  

[CeNi_6_(C_3_H_6_NO_2_)_12_][Ce(NO_3_)_3_(OH)_3_(H_2_O)]
*M* *_r_* = 1944.63
Trigonal, 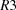

*a* = 14.6418 (4) Å
*c* = 31.7767 (19) Å
*V* = 5899.7 (6) Å^3^
*Z* = 3
Mo *K*α radiation
μ = 2.62 mm^−1^
*T* = 150 K
0.40 × 0.40 × 0.40 mm

### Data collection  

Bruker APEXII CCD diffractometer
Absorption correction: multi-scan (*SADABS*; Bruker, 2008 ▸) *T*_min_ = 0.420, *T*_max_ = 0.420
21829 measured reflections
6978 independent reflections
6734 reflections with *I* > 2σ(*I*)
*R*_int_ = 0.021

### Refinement  

*R*[*F* ^2^ > 2σ(*F* ^2^)] = 0.025
*wR*(*F* ^2^) = 0.066
*S* = 1.02
6978 reflections
327 parameters
1 restraint
H-atom parameters constrained
Δρ_max_ = 0.57 e Å^−3^
Δρ_min_ = −0.69 e Å^−3^
Absolute structure: Flack (1983 ▸), 3493 Friedel pairs
Absolute structure parameter: −0.012 (11)

### 

Data collection: *APEX2* (Bruker, 2008 ▸); cell refinement: *SAINT* (Bruker, 2008 ▸); data reduction: *SAINT*; program(s) used to solve structure: *SHELXTL* (Sheldrick, 2008 ▸); program(s) used to refine structure: *SHELXTL*; molecular graphics: *SHELXTL*; software used to prepare material for publication: *SHELXTL*.

## Supplementary Material

Crystal structure: contains datablock(s) I. DOI: 10.1107/S2056989015017132/wm5213sup1.cif

Structure factors: contains datablock(s) I. DOI: 10.1107/S2056989015017132/wm5213Isup2.hkl

Supporting information file. DOI: 10.1107/S2056989015017132/wm5213Isup3.mol

**Fig. 1.**
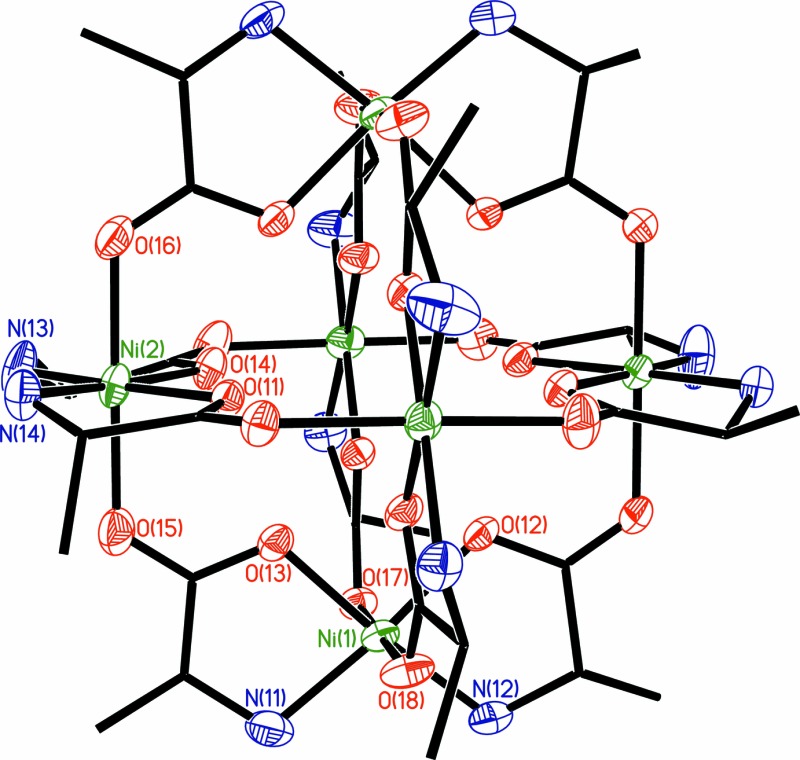
The molecular structure of the {Ni(ala)2}6 unit of the cation (ala = deprotonated L-alanine). Displacement ellipsoids are shown at the 50% probability level. Hydrogen atoms are omitted for clarity.

**Fig. 2.**
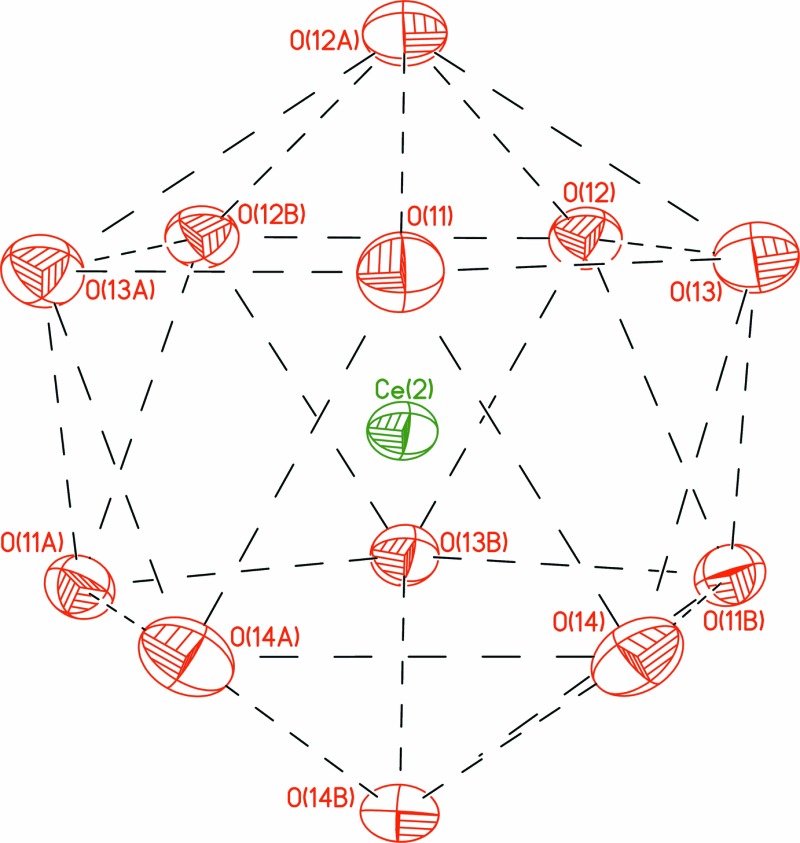
The coordination polyhedron of Ce2 in the complex cation of the title compound. Displacement ellipsoids are shown at the 50% probability level. A and B indicate symmetry operators -y + 3, x - y + 4, z and -x + y, -x + 3, z, respectively.

2 6 l . DOI: 10.1107/S2056989015017132/wm5213fig1.tif
The mol­ecular structure of the {Ni(ala)_2_}_6_ unit of the cation (ala = deprotonated l-alanine). Displacement ellipsoids are shown at the 50% probability level. Hydrogen atoms are omitted for clarity.

y x y z x y x z . DOI: 10.1107/S2056989015017132/wm5213fig2.tif
The coordination polyhedron of Ce2 in the complex cation of the title compound. Displacement ellipsoids are shown at the 50% probability level. A and B indicate symmetry operators −*y* + 3, *x* − *y* + 4, *z* and −*x* + *y*, −*x* + 3, *z*, respectively.

**Fig. 3.**
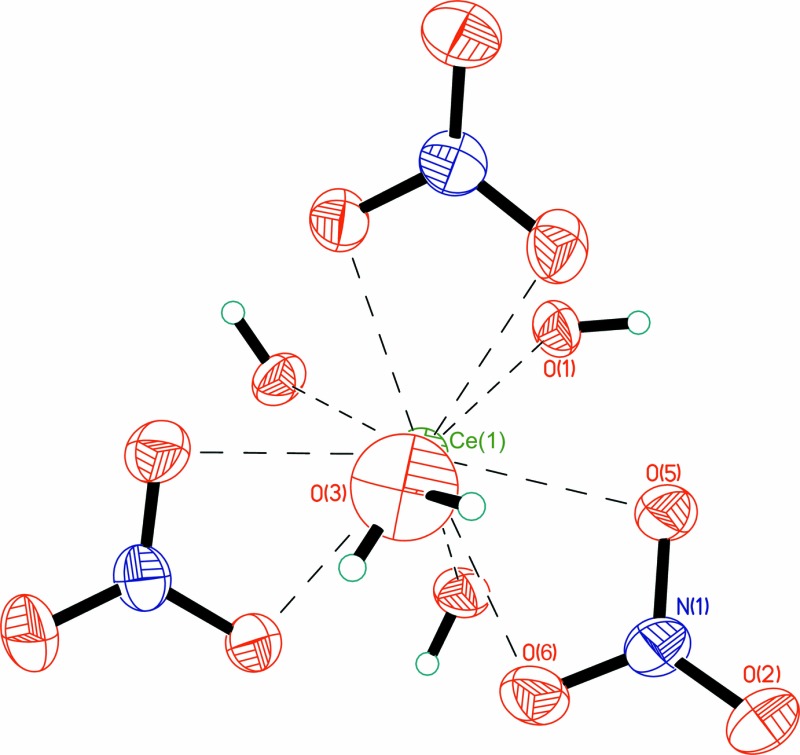
The structure of the complex anion [Ce(NO3)3(OH)3(H2O)]3- in the title compound. Displacement ellipsoids are shown at the 50% probability level. Only one of the orientations of the water molecule is shown.

3 3 3 2 3− . DOI: 10.1107/S2056989015017132/wm5213fig3.tif
The structure of the complex anion [Ce(NO_3_)_3_(OH)_3_(H_2_O)]^3−^ in the title compound. Displacement ellipsoids are shown at the 50% probability level. Only one of the orientations of the water mol­ecule is shown.

**Fig. 4.**
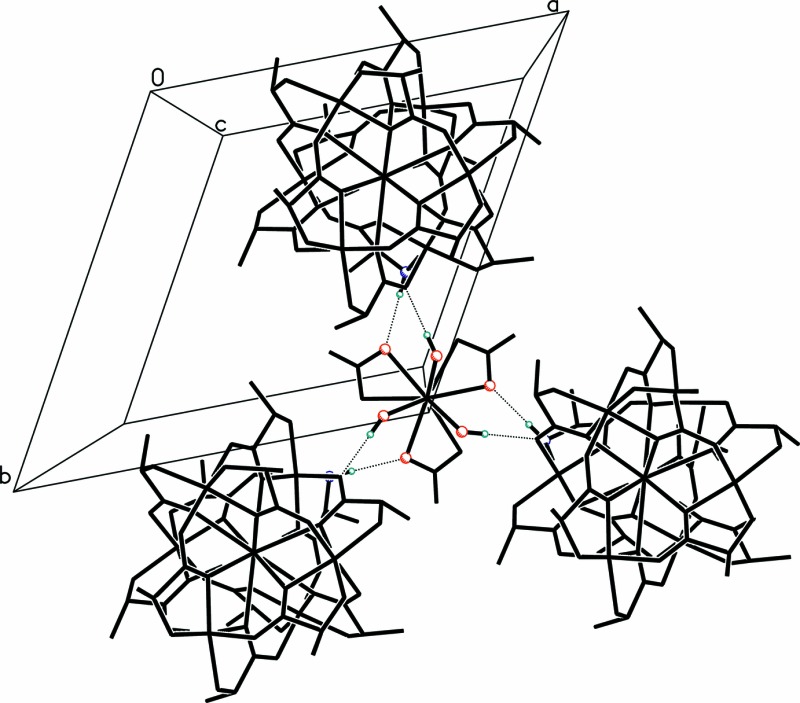
Hydrogen-bonding interactions (dotted lines) between the anion and cations.

. DOI: 10.1107/S2056989015017132/wm5213fig4.tif
Hydrogen-bonding inter­actions (dotted lines) between the anion and cations.

CCDC reference: 1421600

Additional supporting information:  crystallographic information; 3D view; checkCIF report

## Figures and Tables

**Table 1:** Hydrogen-bond geometry (, )

*D*H*A*	*D*H	H*A*	*D* *A*	*D*H*A*
O1H1O17^i^	0.85	1.93	2.758(3)	165
N11H11*B*O6^ii^	0.92	2.38	3.158(5)	143
N12H12*D*O5^iii^	0.92	2.17	3.086(4)	174
N13H13*B*O2^iv^	0.92	2.66	3.284(4)	126

Symmetry codes: (i) 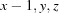; (ii) 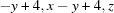; (iii) 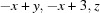; (iv) 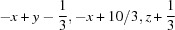.

## supplementary crystallographic information

### S1. Experimental

Crystals of the title complex were obtained in the course of several days after addition of a Ce(NO_3_)_3_ solution in a water-ethanol-methanol mixture to an aquous solution of Ni^II^*L*-alaninate.

### S2. Refinement

The title complex crystallizes in a chiral space group due to the presence of optically pure *L*-alanine in the cation.

A region of electron density was treated with the SQUEEZE procedure in *PLATON* (Spek, 2015). The total potential solvent-accessible void volume is 1020.6 Å^3^, with an estimated electron count of 437. This accounts to approximately 12–15 disordered solvent ethanol and 6–9 water molecules. Their contributions to the total intensity data were removed. The given chemical formula and other crystal data do not take into account the amount of the unknown solvent molecules.

A part of the *L*-alaninato ligands were found to be disordered over two sets of sites with refined component ratios of 0.608 (14):0.392 (14) for the (C4—C6)/(C41—C61) fragment and 0.669 (8):0.331 (8) for the (C11—C12)/(C21—C22) fragment. Disorder was also observed for the coordinating water molecule (O3) situated on a threefold rotation axis. Owing to symmetry restraints the attached hydrogen atoms are disordered over three sites with an occupancy of one-thirds each.

Hydrogen atoms involved in hydrogen bonds (H1, H11B, H12D, and H13B) were located from difference maps and refined using a riding model, with O—H = 0.85 Å and *U*_iso_(H) = 1.5*U*_eq_(O), N—H = 0.92 Å and *U*_iso_(H) = 1.2*U*_eq_(N). All other hydrogen atoms were placed in calculated positions and refined using a riding model with C—H = 0.98 –1.00 Å and *U*_iso_(H) = 1.5*U*_eq_(C*H*_3_), 1.2*U*_eq_(C*H*).

### Crystal data

**Table:**

[CeNi_6_(C_3_H_6_NO_2_)_12_][Ce(NO_3_)_3_(OH)_3_(H_2_O)]	*D*_x_ = 1.642 Mg m^−^^3^
*M**_r_* = 1944.63	Mo *K*α radiation, λ = 0.71073 Å
Trigonal, *R*3	Cell parameters from 9898 reflections
Hall symbol: R 3	θ = 2.3–30.5°
*a* = 14.6418 (4) Å	µ = 2.62 mm^−^^1^
*c* = 31.7767 (19) Å	*T* = 150 K
*V* = 5899.7 (6) Å^3^	Prism, violet
*Z* = 3	0.40 × 0.40 × 0.40 mm
*F*(000) = 2934	

### Data collection

**Table:**

Bruker APEXII CCD diffractometer	6978 independent reflections
Radiation source: fine-focus sealed tube	6734 reflections with *I* > 2σ(*I*)
Graphite monochromator	*R*_int_ = 0.021
ω scans	θ_max_ = 29.0°, θ_min_ = 1.7°
Absorption correction: multi-scan (*SADABS*; Bruker, 2008)	*h* = −19→19
*T*_min_ = 0.420, *T*_max_ = 0.420	*k* = −19→19
21829 measured reflections	*l* = −43→43

### Refinement

**Table:**

Refinement on *F*^2^	Secondary atom site location: difference Fourier map
Least-squares matrix: full	Hydrogen site location: inferred from neighbouring sites
*R*[*F*^2^ > 2σ(*F*^2^)] = 0.025	H-atom parameters constrained
*wR*(*F*^2^) = 0.066	*w* = 1/[σ^2^(*F*_o_^2^) + (0.0434*P*)^2^ + 4.4547*P*] where *P* = (*F*_o_^2^ + 2*F*_c_^2^)/3
*S* = 1.02	(Δ/σ)_max_ = 0.003
6978 reflections	Δρ_max_ = 0.57 e Å^−^^3^
327 parameters	Δρ_min_ = −0.69 e Å^−^^3^
1 restraint	Absolute structure: Flack (1983), 3493 Friedel pairs
Primary atom site location: structure-invariant direct methods	Absolute structure parameter: −0.012 (11)

### Special details

**Table:**

Geometry. All e.s.d.'s (except the e.s.d. in the dihedral angle between two l.s. planes) are estimated using the full covariance matrix. The cell e.s.d.'s are taken into account individually in the estimation of e.s.d.'s in distances, angles and torsion angles; correlations between e.s.d.'s in cell parameters are only used when they are defined by crystal symmetry. An approximate (isotropic) treatment of cell e.s.d.'s is used for estimating e.s.d.'s involving l.s. planes.
Refinement. Refinement of *F*^2^ against ALL reflections. The weighted *R*-factor *wR* and goodness of fit *S* are based on *F*^2^, conventional *R*-factors *R* are based on *F*, with *F* set to zero for negative *F*^2^. The threshold expression of *F*^2^ > σ(*F*^2^) is used only for calculating *R*-factors(gt) *etc*. and is not relevant to the choice of reflections for refinement. *R*-factors based on *F*^2^ are statistically about twice as large as those based on *F*, and *R*- factors based on ALL data will be even larger.

### Fractional atomic coordinates and isotropic or equivalent isotropic displacement parameters (Å^2^)

**Table:**

	*x*	*y*	*z*	*U*_iso_*/*U*_eq_	Occ. (<1)
Ce1	0.6667	2.3333	−1.143417 (9)	0.03086 (6)	
Ce2	1.0000	2.0000	−1.058354 (6)	0.02110 (5)	
Ni1	1.22355 (3)	2.18294 (3)	−1.125638 (10)	0.02623 (8)	
Ni2	1.04234 (3)	2.22458 (3)	−0.991516 (11)	0.03043 (9)	
O1	0.53783 (17)	2.31437 (18)	−1.08711 (7)	0.0354 (5)	
H1	0.4768	2.2597	−1.0881	0.053*	
O2	0.9922 (2)	2.4685 (3)	−1.17180 (10)	0.0583 (8)	
O3	0.6667	2.3333	−1.22069 (16)	0.0798 (17)	
H3	0.7213	2.3777	−1.2341	0.096*	0.3333
H2	0.6121	2.2889	−1.2341	0.096*	0.3333
O5	0.83829 (19)	2.33357 (19)	−1.15778 (8)	0.0441 (5)	
O6	0.8541 (2)	2.4859 (2)	−1.17100 (9)	0.0484 (6)	
O11	0.95267 (16)	2.15480 (15)	−1.04388 (6)	0.0252 (4)	
O12	1.12234 (16)	2.02412 (17)	−1.12577 (6)	0.0268 (4)	
O13	1.15557 (16)	2.20363 (16)	−1.07328 (6)	0.0274 (4)	
O14	1.09798 (16)	2.12172 (17)	−0.99153 (6)	0.0299 (4)	
O15	1.1670 (2)	2.3264 (2)	−1.02855 (8)	0.0402 (6)	
O16	0.9167 (2)	2.1220 (2)	−0.95514 (7)	0.0446 (6)	
O17	1.32725 (16)	2.16186 (16)	−1.08770 (6)	0.0286 (4)	
O18	1.12072 (18)	2.20121 (19)	−1.16376 (7)	0.0362 (5)	
N1	0.8977 (2)	2.4307 (2)	−1.16730 (9)	0.0407 (6)	
N11	1.3169 (3)	2.3429 (2)	−1.12060 (12)	0.0541 (9)	
H11B	1.3868	2.3617	−1.1229	0.065*	
H11C	1.3012	2.3757	−1.1418	0.065*	
N12	1.2828 (2)	2.1486 (2)	−1.17775 (8)	0.0328 (5)	
H12E	1.2995	2.1995	−1.1980	0.039*	
H12D	1.3432	2.1473	−1.1708	0.039*	
N13	1.1359 (3)	2.2836 (3)	−0.93873 (9)	0.0460 (7)	
H13A	1.1661	2.3558	−0.9378	0.055*	
H13B	1.0962	2.2556	−0.9148	0.055*	
N14	0.9681 (2)	2.3129 (2)	−0.99455 (9)	0.0375 (6)	
H14A	0.9169	2.2909	−0.9741	0.045*	
H14B	1.0163	2.3829	−0.9899	0.045*	
C1	1.1303 (2)	1.9766 (2)	−1.15825 (9)	0.0290 (5)	
C2	1.2482 (4)	1.9884 (4)	−1.21822 (13)	0.0582 (12)	
H2A	1.2934	2.0342	−1.2409	0.087*	
H2B	1.1908	1.9234	−1.2303	0.087*	
H2C	1.2900	1.9710	−1.1992	0.087*	
C3	1.2025 (3)	2.0449 (3)	−1.19394 (9)	0.0370 (7)	
H3A	1.1575	2.0578	−1.2140	0.044*	
C4	1.3104 (6)	2.4840 (5)	−1.0800 (4)	0.069 (3)	0.608 (14)
H4A	1.3773	2.5341	−1.0934	0.104*	0.608 (14)
H4B	1.3104	2.5057	−1.0508	0.104*	0.608 (14)
H4C	1.2519	2.4830	−1.0953	0.104*	0.608 (14)
C6	1.2977 (9)	2.3741 (8)	−1.0806 (4)	0.0300 (17)	0.608 (14)
H6A	1.3546	2.3787	−1.0617	0.036*	0.608 (14)
C41	1.2506 (9)	2.4577 (8)	−1.1104 (4)	0.053 (3)	0.392 (14)
H41A	1.3097	2.5160	−1.1252	0.080*	0.392 (14)
H41B	1.2183	2.4858	−1.0912	0.080*	0.392 (14)
H41C	1.1980	2.4109	−1.1309	0.080*	0.392 (14)
C61	1.2894 (14)	2.3979 (13)	−1.0862 (6)	0.034 (3)	0.392 (14)
H61A	1.3514	2.4445	−1.0682	0.041*	0.392 (14)
C5	0.9200 (3)	2.3002 (2)	−1.03646 (10)	0.0339 (6)	
H5A	0.8506	2.2970	−1.0332	0.041*	
C7	0.9018 (2)	2.1977 (2)	−1.05742 (9)	0.0256 (5)	
C8	1.1977 (2)	2.2971 (2)	−1.06015 (10)	0.0327 (6)	
C9	0.9906 (3)	2.3928 (3)	−1.06485 (13)	0.0509 (9)	
H9A	1.0024	2.4586	−1.0518	0.076*	
H9B	0.9564	2.3839	−1.0922	0.076*	
H9C	1.0583	2.3956	−1.0687	0.076*	
C10	0.8608 (4)	2.0257 (3)	−0.96288 (11)	0.0478 (9)	
C11	1.2173 (4)	2.2543 (4)	−0.94147 (14)	0.0377 (12)	0.669 (8)
H11A	1.2765	2.3064	−0.9595	0.045*	0.669 (8)
C12	1.2588 (6)	2.2508 (6)	−0.89832 (19)	0.0550 (18)	0.669 (8)
H12A	1.3132	2.2308	−0.9010	0.082*	0.669 (8)
H12B	1.2009	2.1989	−0.8809	0.082*	0.669 (8)
H12C	1.2892	2.3205	−0.8852	0.082*	0.669 (8)
C21	1.1527 (10)	2.1841 (9)	−0.9212 (3)	0.041 (3)	0.331 (8)
H21A	1.2253	2.2204	−0.9089	0.049*	0.331 (8)
C22	1.0844 (12)	2.1161 (9)	−0.8863 (3)	0.056 (3)	0.331 (8)
H22A	1.1039	2.0628	−0.8794	0.084*	0.331 (8)
H22B	1.0103	2.0810	−0.8951	0.084*	0.331 (8)
H22C	1.0940	2.1597	−0.8615	0.084*	0.331 (8)

### Atomic displacement parameters (Å^2^)

**Table:**

	*U*^11^	*U*^22^	*U*^33^	*U*^12^	*U*^13^	*U*^23^
Ce1	0.03464 (9)	0.03464 (9)	0.02330 (11)	0.01732 (4)	0.000	0.000
Ce2	0.02472 (7)	0.02472 (7)	0.01385 (9)	0.01236 (3)	0.000	0.000
Ni1	0.02819 (18)	0.03022 (17)	0.02059 (16)	0.01484 (14)	0.00288 (13)	0.00459 (13)
Ni2	0.0361 (2)	0.0351 (2)	0.02166 (16)	0.01905 (16)	−0.00561 (13)	−0.01041 (14)
O1	0.0299 (10)	0.0360 (11)	0.0344 (11)	0.0120 (9)	0.0075 (8)	−0.0004 (9)
O2	0.0441 (15)	0.0612 (17)	0.0574 (17)	0.0173 (13)	0.0195 (13)	0.0173 (14)
O3	0.106 (3)	0.106 (3)	0.027 (2)	0.0530 (14)	0.000	0.000
O5	0.0432 (12)	0.0381 (11)	0.0496 (13)	0.0192 (10)	0.0168 (10)	0.0074 (10)
O6	0.0564 (15)	0.0383 (12)	0.0498 (14)	0.0231 (12)	0.0179 (12)	0.0144 (11)
O11	0.0291 (9)	0.0269 (9)	0.0205 (9)	0.0147 (8)	−0.0004 (7)	−0.0026 (7)
O12	0.0302 (10)	0.0337 (10)	0.0165 (8)	0.0160 (9)	0.0014 (7)	0.0002 (7)
O13	0.0325 (10)	0.0319 (10)	0.0203 (9)	0.0179 (8)	−0.0003 (7)	0.0001 (7)
O14	0.0341 (10)	0.0383 (11)	0.0199 (9)	0.0201 (9)	−0.0066 (8)	−0.0077 (8)
O15	0.0399 (13)	0.0391 (13)	0.0344 (12)	0.0144 (10)	−0.0044 (10)	−0.0138 (10)
O16	0.0693 (17)	0.0407 (13)	0.0265 (10)	0.0297 (13)	0.0145 (11)	0.0006 (9)
O17	0.0254 (9)	0.0325 (10)	0.0255 (9)	0.0127 (8)	0.0000 (8)	0.0033 (8)
O18	0.0357 (11)	0.0485 (13)	0.0271 (10)	0.0231 (10)	0.0071 (9)	0.0162 (9)
N1	0.0426 (14)	0.0381 (14)	0.0331 (13)	0.0139 (12)	0.0149 (11)	0.0092 (10)
N11	0.067 (2)	0.0319 (15)	0.059 (2)	0.0219 (15)	0.0316 (17)	0.0141 (14)
N12	0.0294 (12)	0.0392 (13)	0.0273 (12)	0.0154 (11)	0.0064 (9)	0.0074 (10)
N13	0.0585 (18)	0.0634 (19)	0.0288 (13)	0.0398 (16)	−0.0168 (12)	−0.0241 (13)
N14	0.0449 (15)	0.0342 (13)	0.0348 (13)	0.0209 (12)	−0.0044 (11)	−0.0103 (11)
C1	0.0288 (13)	0.0352 (14)	0.0177 (11)	0.0121 (11)	0.0011 (9)	−0.0003 (10)
C2	0.055 (2)	0.060 (2)	0.037 (2)	0.0111 (19)	0.0209 (17)	−0.0129 (17)
C3	0.0332 (14)	0.0494 (18)	0.0184 (12)	0.0131 (13)	0.0031 (10)	−0.0014 (12)
C4	0.044 (4)	0.029 (3)	0.126 (8)	0.010 (3)	0.020 (4)	−0.002 (4)
C6	0.034 (3)	0.015 (4)	0.040 (4)	0.011 (3)	−0.003 (3)	−0.006 (3)
C41	0.051 (6)	0.040 (5)	0.071 (8)	0.024 (5)	0.016 (5)	0.018 (5)
C61	0.032 (5)	0.017 (7)	0.047 (6)	0.007 (4)	0.001 (4)	0.005 (5)
C5	0.0357 (15)	0.0308 (14)	0.0352 (15)	0.0167 (12)	−0.0037 (12)	−0.0101 (12)
C7	0.0240 (12)	0.0240 (12)	0.0252 (12)	0.0094 (10)	0.0020 (9)	−0.0014 (10)
C8	0.0288 (14)	0.0334 (14)	0.0324 (14)	0.0129 (12)	−0.0075 (11)	−0.0071 (12)
C9	0.060 (2)	0.0285 (15)	0.053 (2)	0.0138 (15)	−0.0117 (17)	0.0019 (14)
C10	0.077 (3)	0.0445 (18)	0.0289 (15)	0.0355 (19)	0.0287 (16)	0.0066 (13)
C11	0.034 (2)	0.051 (3)	0.028 (2)	0.021 (2)	−0.0061 (17)	−0.019 (2)
C12	0.069 (4)	0.080 (4)	0.040 (3)	0.055 (4)	−0.032 (3)	−0.032 (3)
C21	0.061 (7)	0.045 (6)	0.034 (5)	0.040 (6)	−0.016 (5)	−0.013 (4)
C22	0.088 (9)	0.046 (6)	0.025 (5)	0.027 (6)	−0.013 (5)	−0.004 (4)

### Geometric parameters (Å, º)

**Table:**

Ce1—O3	2.455 (5)	N12—C3	1.472 (4)
Ce1—O1^i^	2.513 (2)	N12—H12E	0.9200
Ce1—O1^ii^	2.513 (2)	N12—H12D	0.9200
Ce1—O1	2.513 (2)	N13—C11	1.457 (6)
Ce1—O5^ii^	2.552 (2)	N13—C21	1.689 (11)
Ce1—O5	2.552 (2)	N13—H13A	0.9200
Ce1—O5^i^	2.552 (2)	N13—H13B	0.9200
Ce1—O6^ii^	2.675 (3)	N14—C5	1.474 (4)
Ce1—O6^i^	2.675 (3)	N14—H14A	0.9200
Ce1—O6	2.675 (3)	N14—H14B	0.9200
Ce2—O14^iii^	2.6808 (19)	C1—O18^iii^	1.246 (4)
Ce2—O14^iv^	2.6808 (19)	C1—C3	1.532 (4)
Ce2—O14	2.6809 (19)	C2—C3	1.510 (5)
Ce2—O12	2.700 (2)	C2—H2A	0.9800
Ce2—O12^iii^	2.700 (2)	C2—H2B	0.9800
Ce2—O12^iv^	2.700 (2)	C2—H2C	0.9800
Ce2—O11^iii^	2.7202 (19)	C3—H3A	1.0000
Ce2—O11	2.720 (2)	C4—C6	1.525 (11)
Ce2—O11^iv^	2.720 (2)	C4—H4A	0.9800
Ce2—O13^iii^	2.741 (2)	C4—H4B	0.9800
Ce2—O13	2.741 (2)	C4—H4C	0.9800
Ce2—O13^iv^	2.741 (2)	C6—C8	1.478 (12)
Ni1—O13	2.038 (2)	C6—H6A	1.0000
Ni1—O12	2.039 (2)	C41—C61	1.476 (17)
Ni1—N11	2.044 (3)	C41—H41A	0.9800
Ni1—N12	2.045 (3)	C41—H41B	0.9800
Ni1—O18	2.051 (2)	C41—H41C	0.9800
Ni1—O17	2.079 (2)	C61—C8	1.638 (17)
Ni2—O14	2.039 (2)	C61—H61A	1.0000
Ni2—O11	2.048 (2)	C5—C9	1.523 (5)
Ni2—O16	2.053 (3)	C5—C7	1.537 (4)
Ni2—O15	2.054 (3)	C5—H5A	1.0000
Ni2—N13	2.062 (3)	C7—O17^iv^	1.256 (4)
Ni2—N14	2.066 (3)	C9—H9A	0.9800
O1—H1	0.8512	C9—H9B	0.9800
O2—N1	1.214 (4)	C9—H9C	0.9800
O3—H3	0.8501	C10—O14^iv^	1.267 (4)
O3—H2	0.8501	C10—C21^iv^	1.528 (11)
O5—N1	1.278 (4)	C10—C11^iv^	1.612 (6)
O6—N1	1.262 (4)	C11—C12	1.511 (7)
O11—C7	1.267 (3)	C11—C10^iii^	1.612 (6)
O12—C1	1.282 (3)	C11—H11A	1.0000
O13—C8	1.259 (4)	C12—H12A	0.9800
O14—C10^iii^	1.266 (4)	C12—H12B	0.9800
O15—C8	1.259 (4)	C12—H12C	0.9800
O16—C10	1.251 (5)	C21—C22	1.493 (18)
O17—C7^iii^	1.256 (4)	C21—C10^iii^	1.528 (10)
O18—C1^iv^	1.247 (4)	C21—H21A	1.0000
N11—C6	1.427 (12)	C22—H22A	0.9800
N11—C61	1.53 (2)	C22—H22B	0.9800
N11—H11B	0.9200	C22—H22C	0.9800
N11—H11C	0.9200		
			
O3—Ce1—O1^i^	135.41 (5)	H3—O3—H2	120.0
O3—Ce1—O1^ii^	135.41 (6)	N1—O5—Ce1	99.56 (19)
O1^i^—Ce1—O1^ii^	74.89 (8)	N1—O6—Ce1	94.10 (17)
O3—Ce1—O1	135.41 (5)	C7—O11—Ni2	114.71 (17)
O1^i^—Ce1—O1	74.89 (8)	C7—O11—Ce2	144.52 (17)
O1^ii^—Ce1—O1	74.89 (8)	Ni2—O11—Ce2	100.73 (8)
O3—Ce1—O5^ii^	79.70 (6)	C1—O12—Ni1	114.16 (18)
O1^i^—Ce1—O5^ii^	82.30 (8)	C1—O12—Ce2	143.31 (18)
O1^ii^—Ce1—O5^ii^	144.27 (8)	Ni1—O12—Ce2	101.76 (8)
O1—Ce1—O5^ii^	72.80 (8)	C8—O13—Ni1	114.99 (19)
O3—Ce1—O5	79.70 (6)	C8—O13—Ce2	144.54 (19)
O1^i^—Ce1—O5	72.80 (8)	Ni1—O13—Ce2	100.46 (8)
O1^ii^—Ce1—O5	82.30 (8)	C10^iii^—O14—Ni2	113.9 (2)
O1—Ce1—O5	144.27 (8)	C10^iii^—O14—Ce2	143.8 (2)
O5^ii^—Ce1—O5	116.87 (4)	Ni2—O14—Ce2	102.27 (8)
O3—Ce1—O5^i^	79.70 (6)	C8—O15—Ni2	123.2 (2)
O1^i^—Ce1—O5^i^	144.27 (8)	C10—O16—Ni2	123.8 (2)
O1^ii^—Ce1—O5^i^	72.80 (8)	C7^iii^—O17—Ni1	122.13 (18)
O1—Ce1—O5^i^	82.30 (8)	C1^iv^—O18—Ni1	123.56 (18)
O5^ii^—Ce1—O5^i^	116.87 (4)	O2—N1—O6	121.5 (3)
O5—Ce1—O5^i^	116.88 (4)	O2—N1—O5	121.5 (3)
O3—Ce1—O6^ii^	70.88 (6)	O6—N1—O5	117.0 (3)
O1^i^—Ce1—O6^ii^	66.55 (8)	C6—N11—C61	17.5 (5)
O1^ii^—Ce1—O6^ii^	136.51 (8)	C6—N11—Ni1	108.1 (5)
O1—Ce1—O6^ii^	111.86 (8)	C61—N11—Ni1	117.3 (7)
O5^ii^—Ce1—O6^ii^	48.87 (8)	C6—N11—H11B	110.1
O5—Ce1—O6^ii^	68.01 (8)	C61—N11—H11B	116.5
O5^i^—Ce1—O6^ii^	148.93 (9)	Ni1—N11—H11B	110.1
O3—Ce1—O6^i^	70.88 (6)	C6—N11—H11C	110.1
O1^i^—Ce1—O6^i^	136.51 (8)	C61—N11—H11C	92.7
O1^ii^—Ce1—O6^i^	111.86 (8)	Ni1—N11—H11C	110.1
O1—Ce1—O6^i^	66.55 (8)	H11B—N11—H11C	108.4
O5^ii^—Ce1—O6^i^	68.01 (8)	C3—N12—Ni1	108.54 (18)
O5—Ce1—O6^i^	148.93 (9)	C3—N12—H12E	110.0
O5^i^—Ce1—O6^i^	48.87 (8)	Ni1—N12—H12E	110.0
O6^ii^—Ce1—O6^i^	109.82 (6)	C3—N12—H12D	110.0
O3—Ce1—O6	70.88 (6)	Ni1—N12—H12D	110.0
O1^i^—Ce1—O6	111.86 (8)	H12E—N12—H12D	108.4
O1^ii^—Ce1—O6	66.55 (8)	C11—N13—C21	43.3 (5)
O1—Ce1—O6	136.51 (8)	C11—N13—Ni2	107.3 (2)
O5^ii^—Ce1—O6	148.93 (9)	C21—N13—Ni2	105.0 (4)
O5—Ce1—O6	48.87 (8)	C11—N13—H13A	110.3
O5^i^—Ce1—O6	68.01 (8)	C21—N13—H13A	141.9
O6^ii^—Ce1—O6	109.82 (6)	Ni2—N13—H13A	110.3
O6^i^—Ce1—O6	109.82 (6)	C11—N13—H13B	110.3
O14^iii^—Ce2—O14^iv^	63.82 (8)	C21—N13—H13B	71.1
O14^iii^—Ce2—O14	63.82 (8)	Ni2—N13—H13B	110.3
O14^iv^—Ce2—O14	63.82 (8)	H13A—N13—H13B	108.5
O14^iii^—Ce2—O12	116.32 (6)	C5—N14—Ni2	109.29 (19)
O14^iv^—Ce2—O12	179.85 (7)	C5—N14—H14A	109.8
O14—Ce2—O12	116.23 (6)	Ni2—N14—H14A	109.8
O14^iii^—Ce2—O12^iii^	116.23 (6)	C5—N14—H14B	109.8
O14^iv^—Ce2—O12^iii^	116.32 (6)	Ni2—N14—H14B	109.8
O14—Ce2—O12^iii^	179.85 (9)	H14A—N14—H14B	108.3
O12—Ce2—O12^iii^	63.62 (7)	O18^iii^—C1—O12	125.0 (3)
O14^iii^—Ce2—O12^iv^	179.85 (7)	O18^iii^—C1—C3	117.4 (3)
O14^iv^—Ce2—O12^iv^	116.23 (6)	O12—C1—C3	117.5 (3)
O14—Ce2—O12^iv^	116.33 (6)	C3—C2—H2A	109.5
O12—Ce2—O12^iv^	63.62 (7)	C3—C2—H2B	109.5
O12^iii^—Ce2—O12^iv^	63.62 (7)	H2A—C2—H2B	109.5
O14^iii^—Ce2—O11^iii^	65.59 (6)	C3—C2—H2C	109.5
O14^iv^—Ce2—O11^iii^	117.85 (6)	H2A—C2—H2C	109.5
O14—Ce2—O11^iii^	62.89 (6)	H2B—C2—H2C	109.5
O12—Ce2—O11^iii^	62.27 (6)	N12—C3—C2	113.6 (3)
O12^iii^—Ce2—O11^iii^	116.99 (6)	N12—C3—C1	110.5 (2)
O12^iv^—Ce2—O11^iii^	114.45 (6)	C2—C3—C1	111.8 (3)
O14^iii^—Ce2—O11	117.85 (6)	N12—C3—H3A	106.9
O14^iv^—Ce2—O11	62.89 (6)	C2—C3—H3A	106.9
O14—Ce2—O11	65.59 (6)	C1—C3—H3A	106.9
O12—Ce2—O11	116.99 (6)	C6—C4—H4A	109.5
O12^iii^—Ce2—O11	114.44 (6)	C6—C4—H4B	109.5
O12^iv^—Ce2—O11	62.27 (6)	H4A—C4—H4B	109.5
O11^iii^—Ce2—O11	117.20 (2)	C6—C4—H4C	109.5
O14^iii^—Ce2—O11^iv^	62.89 (6)	H4A—C4—H4C	109.5
O14^iv^—Ce2—O11^iv^	65.59 (6)	H4B—C4—H4C	109.5
O14—Ce2—O11^iv^	117.85 (6)	N11—C6—C8	115.2 (7)
O12—Ce2—O11^iv^	114.45 (6)	N11—C6—C4	114.2 (8)
O12^iii^—Ce2—O11^iv^	62.27 (6)	C8—C6—C4	110.6 (7)
O12^iv^—Ce2—O11^iv^	116.99 (6)	N11—C6—H6A	105.3
O11^iii^—Ce2—O11^iv^	117.20 (2)	C8—C6—H6A	105.3
O11—Ce2—O11^iv^	117.20 (2)	C4—C6—H6A	105.3
O14^iii^—Ce2—O13^iii^	62.38 (6)	C61—C41—H41A	109.5
O14^iv^—Ce2—O13^iii^	117.36 (6)	C61—C41—H41B	109.5
O14—Ce2—O13^iii^	114.53 (6)	H41A—C41—H41B	109.5
O12—Ce2—O13^iii^	62.76 (6)	C61—C41—H41C	109.5
O12^iii^—Ce2—O13^iii^	65.43 (6)	H41A—C41—H41C	109.5
O12^iv^—Ce2—O13^iii^	117.50 (6)	H41B—C41—H41C	109.5
O11^iii^—Ce2—O13^iii^	62.76 (6)	C41—C61—N11	102.8 (12)
O11—Ce2—O13^iii^	179.74 (7)	C41—C61—C8	114.4 (12)
O11^iv^—Ce2—O13^iii^	62.97 (6)	N11—C61—C8	101.6 (9)
O14^iii^—Ce2—O13	117.36 (6)	C41—C61—H61A	112.4
O14^iv^—Ce2—O13	114.53 (6)	N11—C61—H61A	112.4
O14—Ce2—O13	62.38 (6)	C8—C61—H61A	112.4
O12—Ce2—O13	65.43 (6)	N14—C5—C9	110.7 (3)
O12^iii^—Ce2—O13	117.50 (6)	N14—C5—C7	110.7 (3)
O12^iv^—Ce2—O13	62.76 (6)	C9—C5—C7	108.8 (3)
O11^iii^—Ce2—O13	62.97 (6)	N14—C5—H5A	108.9
O11—Ce2—O13	62.76 (6)	C9—C5—H5A	108.9
O11^iv^—Ce2—O13	179.74 (7)	C7—C5—H5A	108.9
O13^iii^—Ce2—O13	117.07 (2)	O17^iv^—C7—O11	125.7 (3)
O14^iii^—Ce2—O13^iv^	114.53 (6)	O17^iv^—C7—C5	115.7 (2)
O14^iv^—Ce2—O13^iv^	62.38 (6)	O11—C7—C5	118.6 (2)
O14—Ce2—O13^iv^	117.36 (6)	O13—C8—O15	124.8 (3)
O12—Ce2—O13^iv^	117.50 (6)	O13—C8—C6	116.3 (5)
O12^iii^—Ce2—O13^iv^	62.76 (6)	O15—C8—C6	118.4 (5)
O12^iv^—Ce2—O13^iv^	65.43 (6)	O13—C8—C61	123.2 (7)
O11^iii^—Ce2—O13^iv^	179.74 (7)	O15—C8—C61	111.6 (7)
O11—Ce2—O13^iv^	62.97 (6)	C6—C8—C61	15.9 (6)
O11^iv^—Ce2—O13^iv^	62.76 (6)	C5—C9—H9A	109.5
O13^iii^—Ce2—O13^iv^	117.07 (2)	C5—C9—H9B	109.5
O13—Ce2—O13^iv^	117.07 (2)	H9A—C9—H9B	109.5
O13—Ni1—O12	92.34 (8)	C5—C9—H9C	109.5
O13—Ni1—N11	82.07 (11)	H9A—C9—H9C	109.5
O12—Ni1—N11	174.40 (11)	H9B—C9—H9C	109.5
O13—Ni1—N12	175.09 (10)	O16—C10—O14^iv^	125.5 (3)
O12—Ni1—N12	82.76 (10)	O16—C10—C21^iv^	107.9 (4)
N11—Ni1—N12	102.83 (12)	O14^iv^—C10—C21^iv^	117.7 (5)
O13—Ni1—O18	91.03 (9)	O16—C10—C11^iv^	117.9 (3)
O12—Ni1—O18	89.15 (9)	O14^iv^—C10—C11^iv^	115.5 (4)
N11—Ni1—O18	90.61 (15)	C21^iv^—C10—C11^iv^	44.1 (5)
N12—Ni1—O18	89.03 (10)	N13—C11—C12	111.0 (4)
O13—Ni1—O17	89.77 (8)	N13—C11—C10^iii^	107.6 (3)
O12—Ni1—O17	90.01 (8)	C12—C11—C10^iii^	109.7 (5)
N11—Ni1—O17	90.31 (14)	N13—C11—H11A	109.5
N12—Ni1—O17	90.11 (10)	C12—C11—H11A	109.5
O18—Ni1—O17	178.87 (10)	C10^iii^—C11—H11A	109.5
O14—Ni2—O11	91.41 (8)	C11—C12—H12A	109.5
O14—Ni2—O16	90.15 (10)	C11—C12—H12B	109.5
O11—Ni2—O16	88.63 (10)	H12A—C12—H12B	109.5
O14—Ni2—O15	89.88 (10)	C11—C12—H12C	109.5
O11—Ni2—O15	90.69 (9)	H12A—C12—H12C	109.5
O16—Ni2—O15	179.32 (11)	H12B—C12—H12C	109.5
O14—Ni2—N13	83.28 (10)	C22—C21—C10^iii^	122.7 (9)
O11—Ni2—N13	174.65 (11)	C22—C21—N13	118.9 (9)
O16—Ni2—N13	90.76 (13)	C10^iii^—C21—N13	100.7 (6)
O15—Ni2—N13	89.92 (12)	C22—C21—H21A	104.2
O14—Ni2—N14	172.61 (10)	C10^iii^—C21—H21A	104.2
O11—Ni2—N14	82.40 (10)	N13—C21—H21A	104.2
O16—Ni2—N14	85.68 (12)	C21—C22—H22A	109.5
O15—Ni2—N14	94.21 (12)	C21—C22—H22B	109.5
N13—Ni2—N14	102.85 (12)	H22A—C22—H22B	109.5
Ce1—O1—H1	117.0	C21—C22—H22C	109.5
Ce1—O3—H3	120.0	H22A—C22—H22C	109.5
Ce1—O3—H2	120.0	H22B—C22—H22C	109.5

Symmetry codes: (i) −*y*+3, *x*−*y*+4, *z*; (ii) −*x*+*y*−1, −*x*+3, *z*; (iii) −*x*+*y*, −*x*+3, *z*; (iv) −*y*+3, *x*−*y*+3, *z*.

### Hydrogen-bond geometry (Å, º)

**Table:**

*D*—H···*A*	*D*—H	H···*A*	*D*···*A*	*D*—H···*A*
O1—H1···O17^v^	0.85	1.93	2.758 (3)	165
N11—H11*B*···O6^vi^	0.92	2.38	3.158 (5)	143
N12—H12*D*···O5^iii^	0.92	2.17	3.086 (4)	174
N13—H13*B*···O2^vii^	0.92	2.66	3.284 (4)	126

Symmetry codes: (iii) −*x*+*y*, −*x*+3, *z*; (v) *x*−1, *y*, *z*; (vi) −*y*+4, *x*−*y*+4, *z*; (vii) −*x*+*y*−1/3, −*x*+10/3, *z*+1/3.
