# Supplementary figures and images for: Crystal structure of an unknown solvate of dodecakis­(μ2-alaninato-1:2κ2 O:N,O)cerium(III)hexa­nickel(II) aqua­tris­(hydroxido-κO)tris­(nitrato-κ2 O,O′)cerate(III)
Source: Acta Crystallogr E Crystallogr Commun. 2015 Sep 17;71(Pt 10):m183–4. doi: 10.1107/S2056989015017132 (PMC4647412; doi:10.1107/S2056989015017132)

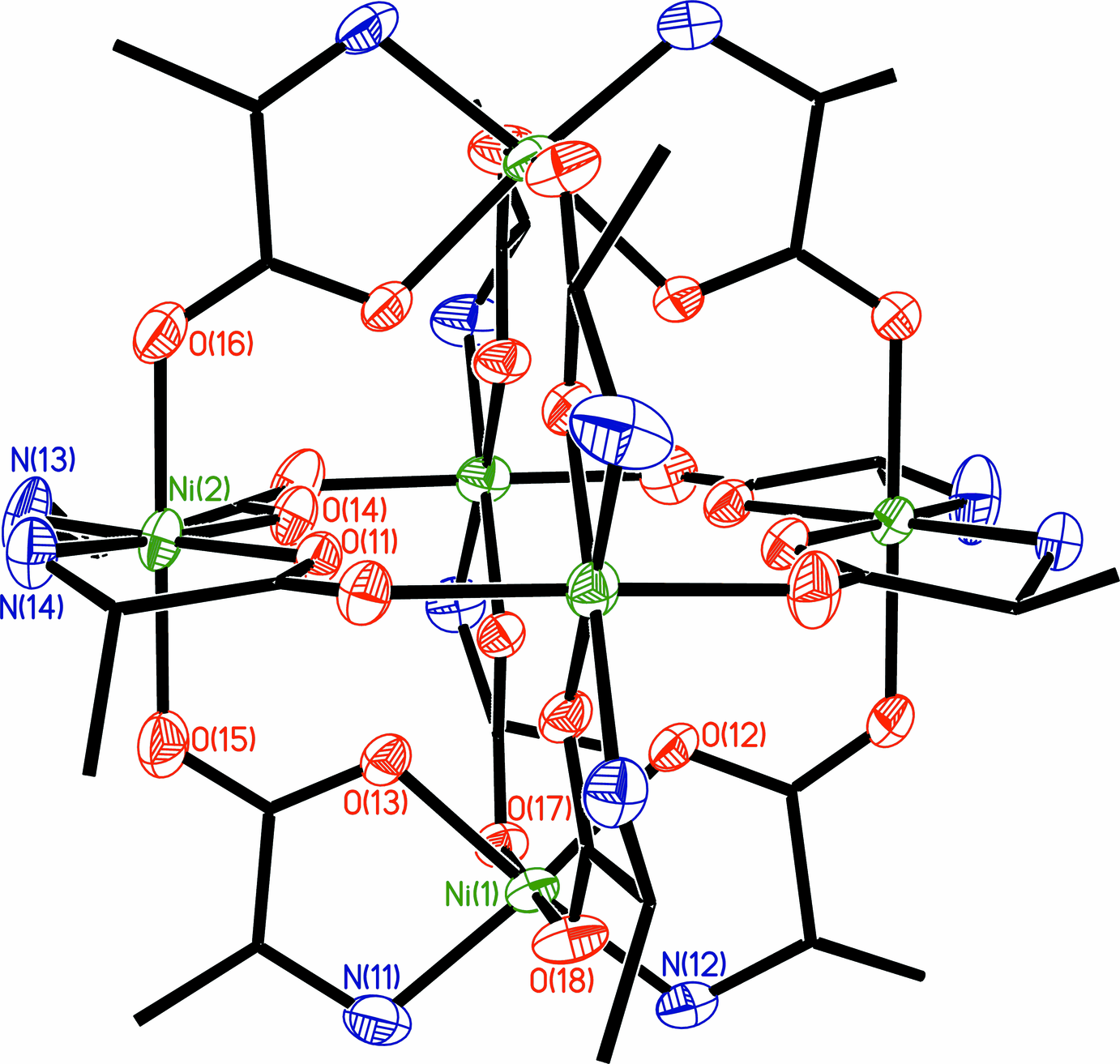

Supplement: Supplementary file 4 [file e-71-0m183-fig1.tif]

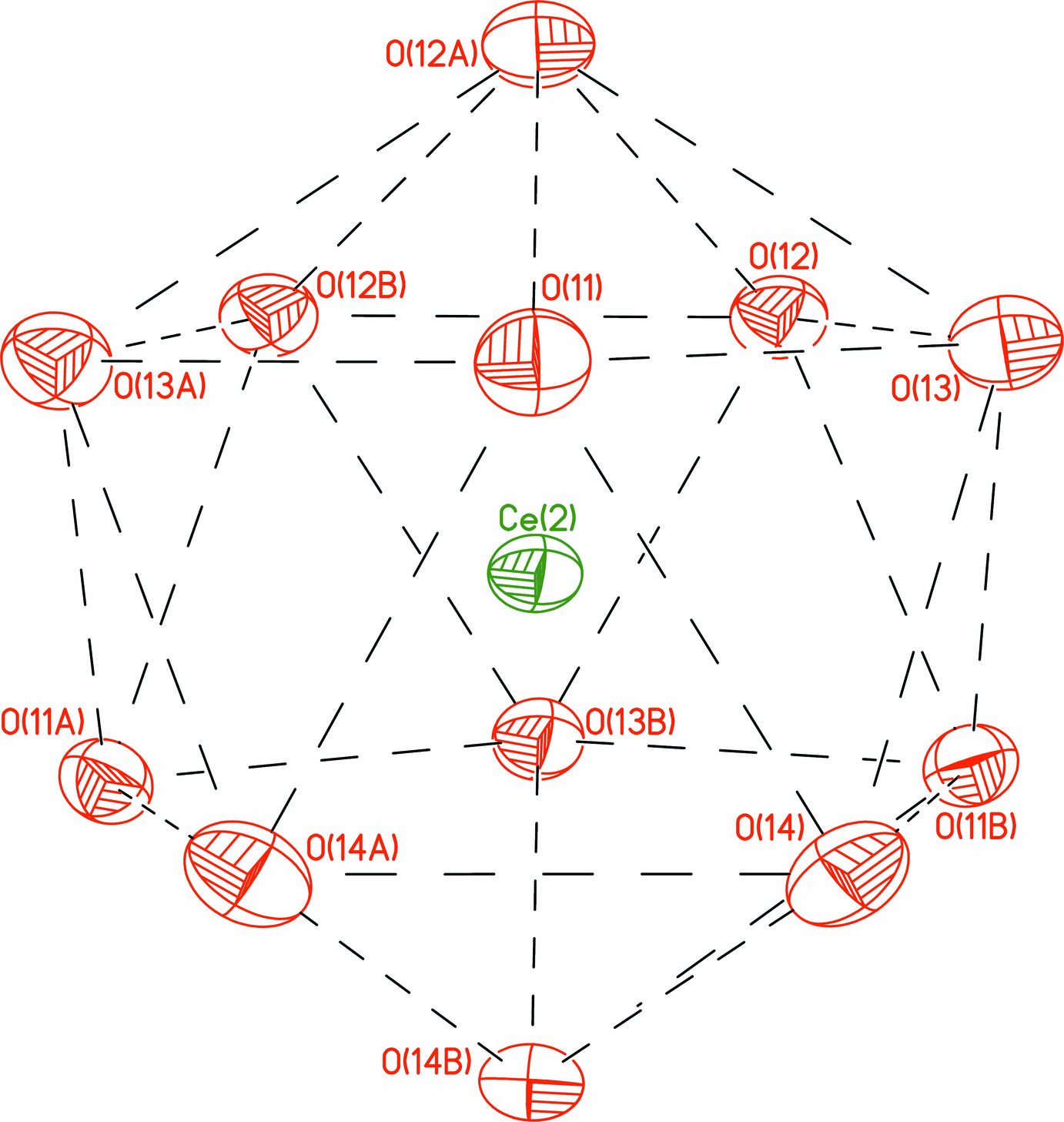

Supplement: Supplementary file 5 [file e-71-0m183-fig2.tif]

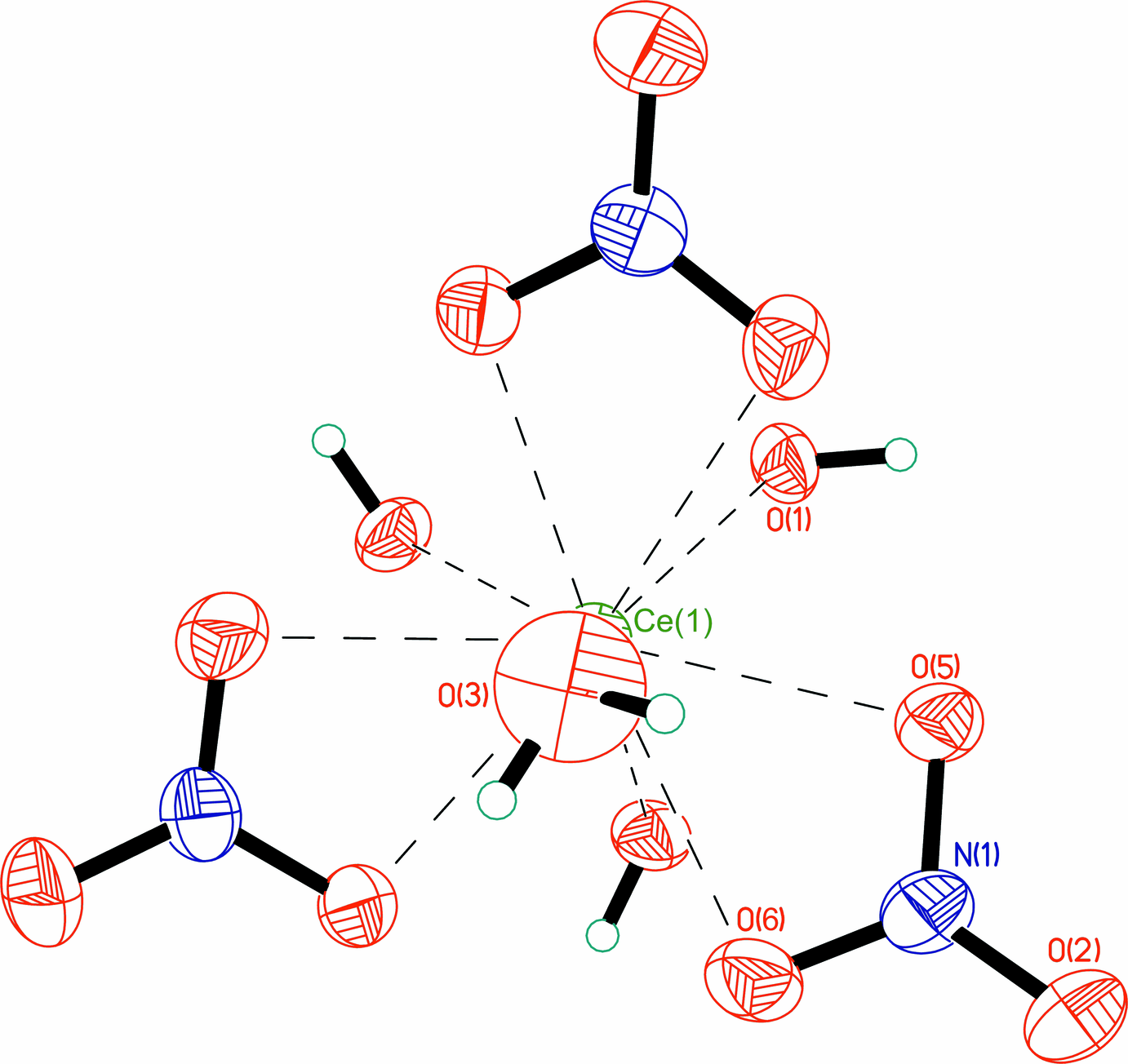

Supplement: Supplementary file 6 [file e-71-0m183-fig3.tif]

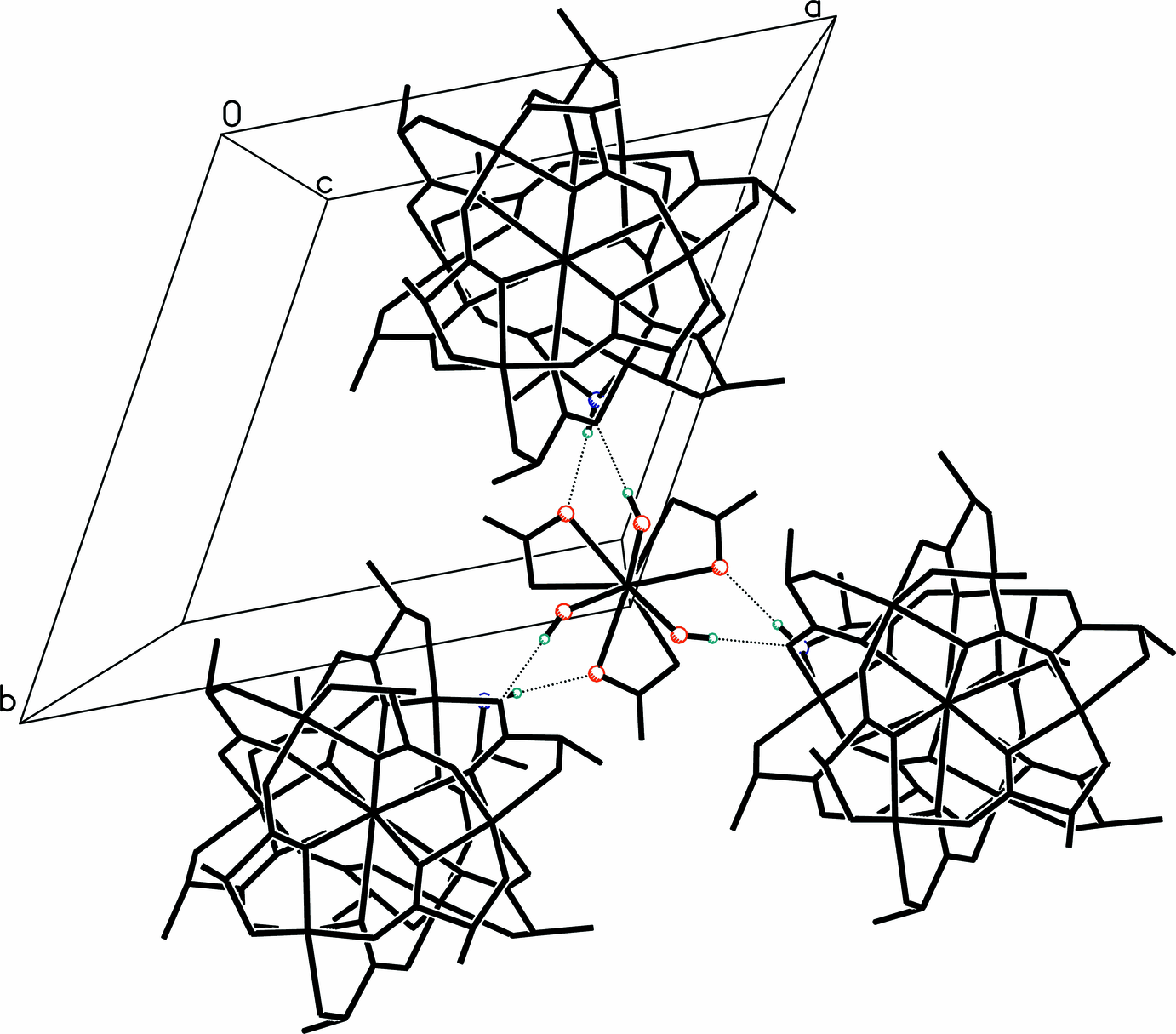

Supplement: Supplementary file 7 [file e-71-0m183-fig4.tif]
